# Simplified Diet for nutrition management of phenylketonuria: A survey of U.S. metabolic dietitians

**DOI:** 10.1002/jmd2.12106

**Published:** 2020-04-08

**Authors:** Joyanna Hansen, Suzanne Hollander, Nicoletta Drilias, Sandra Van Calcar, Fran Rohr, Laurie Bernstein

**Affiliations:** ^1^ Department of Molecular and Medical Genetics Oregon Health & Science University Portland Oregon; ^2^ UCLA Health, UCLA David Geffen School of Medicine Los Angeles California; ^3^ Waisman Center University of Wisconsin – Madison Madison Wisconsin; ^4^ Met Ed Co. Boulder Colorado; ^5^ Department of Pediatrics Section of Clinical Genetics and Metabolism Children's Hospital Colorado, University of Colorado, Anschutz Medical Campus Aurora Colorado

**Keywords:** dietitian, metabolic, phenylketonuria, PKU, simplified diet

## Abstract

**Background:**

Phenylketonuria (PKU) is an inherited metabolic disorder affecting the conversion of phenylalanine (Phe) to tyrosine. Medical nutrition therapy, consisting of a Phe‐restricted diet with medical formula, is the primary treatment for PKU. The Simplified Diet is an approach to PKU nutrition management that allows certain fruits, vegetables, and low‐protein foods to be eaten without measuring or tracking, referred to as free/uncounted foods. There is no consensus on how to implement this approach in metabolic centers in the United States (U.S.), and clinical practice varies.

**Aim:**

This study describes the clinical experience of metabolic dietitians in U.S.‐based metabolic centers related to the use and implementation of the Simplified Diet.

**Methods:**

A survey was developed and sent out to metabolic dietitians to query current clinical practices related to the Simplified Diet. Descriptive statistics were used to analyze responses.

**Results:**

Sixty‐three dietitians managing ≥5 patients with PKU in U.S.‐based metabolic centers responded to the survey. Ninety‐eight percent of survey respondents reported using some version of the Simplified Diet in clinical practice. The survey identified areas of strong agreement, including introduction of the Simplified Diet at 6 to 12 months of age. The survey also identified areas of widespread variability, including specific Phe or protein thresholds for free/uncounted foods, and whether or not to set daily quantity limits on these foods.

**Conclusions:**

Significant variability related to implementation of the Simplified Diet exists across U.S.‐based metabolic centers. This practice variability may contribute to differences in the patient experience across centers and may indicate a need for development of clinical guidelines.

**SYNOPSIS**

Results from a survey of U.S. metabolic dietitians demonstrate variation in clinical experience related to use and implementation of the Simplified Diet for PKU.

## 1 INTRODUCTION

Phenylketonuria (PKU) is a rare inherited metabolic disorder with an incidence of about 1 in 10 000 live births. PKU is caused by pathogenic variants in the phenylalanine hydroxylase (*PAH*) gene, resulting in reduced conversion of the essential amino acid phenylalanine (Phe) into tyrosine.^1^ While several pharmacologic therapies are available,^1^, ^2^ medical nutrition therapy (MNT) is the primary treatment for PKU, and requires restricting dietary Phe to individual Phe tolerance and supplementing with Phe‐free medical foods to meet total protein needs.^3^ Untreated PKU results in severe, permanent intellectual disability. While early treatment can prevent intellectual disability, individuals with PKU who have suboptimal metabolic control later in life can experience adverse neurocognitive outcomes.^1^

Most individuals with PKU have a dietary protein tolerance of less than 10 g/day (~500 mg Phe/day), which necessitates exclusion of meat, dairy, and high‐protein plant‐based foods.^4^ Traditionally, individuals with PKU measured and tracked the Phe content of all foods consumed in order not to exceed their prescribed Phe allowance.^3^ The severe restrictions of the PKU diet, combined with psychosocial barriers, have resulted in poor dietary compliance among individuals with PKU.^5^, ^6^ Identifying strategies to increase adherence to the PKU diet is a significant challenge for both patients and clinicians.

The Simplified Diet is an approach to PKU dietary management that allows for unrestricted intake of foods low in Phe, including many fruits, vegetables, and modified low‐protein medical foods; these foods are considered “free” or “uncounted.”^7^ While the diet is simplified, it is not liberalized in terms of increasing the overall amount of Phe that is allowed; rather, the amount of “counted” Phe allowed in the diet is decreased to offset the small amount of Phe in free/uncounted foods. Typically, an individual's dietary Phe prescription is decreased by approximately 30% to account for intake of Phe from free/uncounted foods, and all other foods with higher protein content continue to be measured and counted towards the daily Phe goal.^7^ The Simplified Diet may promote increased diet flexibility and encourage greater intake of very low‐Phe foods, such as fruits and vegetables, by removing the patient burden of tracking free/uncounted foods.^8^, ^9^, ^10^ The Simplified Diet has been shown to maintain comparable metabolic control compared to patients following a traditional diet where all foods are counted and measured.^7^, ^8^, ^9^, ^10^, ^11^

Clinicians' experiences with “free foods” have been described for European centers^12^; however, no such data exists for metabolic centers in the United States. To address this gap, we report the results of a survey of dietitians in U.S.‐based metabolic centers to describe current use and implementation of the Simplified Diet for PKU.

## 2 MATERIALS AND METHODS

### 2.1 Study design

A survey invitation was sent out to health professionals through an email listserv widely used by metabolic dietitians (GNO‐METAB Listserv, http://genetics.emory.edu/pnometabolic/). The survey included screening questions to identify registered dietitians (RDs) currently employed in a metabolic center in the United States who manage ≥5 patients with PKU. Survey participants who met the screening criteria completed an electronic consent form and the Simplified Diet survey. Participant responses were anonymous. Survey data were collected and managed using REDCap hosted at Oregon Health & Science University.^13^, ^14^ REDCap (Research Electronic Data Capture) is a secure, web‐based software platform designed to support data capture for research studies.

The Simplified Diet survey included 27 questions; key question domains included clinic information/demographics, criteria for defining free/uncounted foods, age of introduction of Simplified Diet, comparison of Simplified Diet to traditional Phe‐counting method, and challenges/barriers related to Simplified Diet implementation. The full questionnaire is available in the online supplement.

The survey was developed by metabolic dietitians with experience using the Simplified Diet in clinical practice. This study was approved by the OHSU Institutional Review Board.

### 2.2 Statistical analysis

Descriptive statistics were performed using SAS (version 9.4) to summarize survey responses and identify areas of agreement and disagreement. GraphPad Prism (version 8) was used to generate figures.

## 3 RESULTS

### 3.1 Study participants

Ninety‐three individuals opened the survey invitation; 12 individuals did not meet the screening criteria (registered dietitian practicing in the United States and managing >5 patients with PKU). An additional 18 individuals met the screening criteria, but did not complete the consent form or did not answer any of the survey questions. Sixty‐three registered dietitians met the screening criteria and completed the survey. A precise response rate cannot be calculated for this survey as the number of practicing metabolic dietitians in the United States is not known; however, for comparison, a professional survey sent out to metabolic dietitians by Genetic Metabolic Dietitians International (GMDI) had a response from about 150 metabolic dietitians in the United States.^15^ In addition, there are 141 U.S.‐based metabolic clinics identified on the GMDI website,^16^ with some clinics employing multiple dietitians. Therefore, survey responses are estimated to represent approximately 30% of U.S.‐based metabolic dietitians in clinical practice.

Participants were distributed geographically across the United States, with representation from the Northeast, Midwest, South, and West regions, and U.S. Territories. Seventy‐one percent of survey participants (n = 45/63) reported working in a public hospital or university medical center, and 54% (n = 34/63) reported working as a metabolic dietitian for ≤5 years. Approximately half of participants (n = 32/63, 51%) reported seeing between 5 and 99 patients with PKU annually in clinic (Table 1).

**Table 1:** Demographic and practice characteristics of survey respondents (n = 63)

Characteristic	n	%
*Geographic region of the United States* a		
Northeast	12	19
Midwest	17	27
South	19	30
West	13	21
U.S. territories	2	3
*Years in practice*		
0‐5	34	54
6‐15	16	25
16+	13	21
*Metabolic clinic setting*		
Public hospital/University medical center	45	71
Private hospital/Medical facility	7	11
Public hospital/Medical facility	10	16
Other	1	2
*Number of PKU patients seen in clinic*		
5‐99	32	51
100‐200	24	38
200+	7	11

a Geographical regions of Northeast, Midwest, South, and West based on U.S. Census Bureau definitions.

### 3.2 Simplified Diet use and phenylalanine/protein thresholds for free/uncounted foods

Ninety‐eight percent of survey respondents (n = 62/63) reported using some version of the Simplified Diet in clinical practice. One participant reported not using any version of the Simplified Diet in clinical practice, and was excluded from questions specifically asking about implementation of the Simplified Diet.

**Figure 1:**
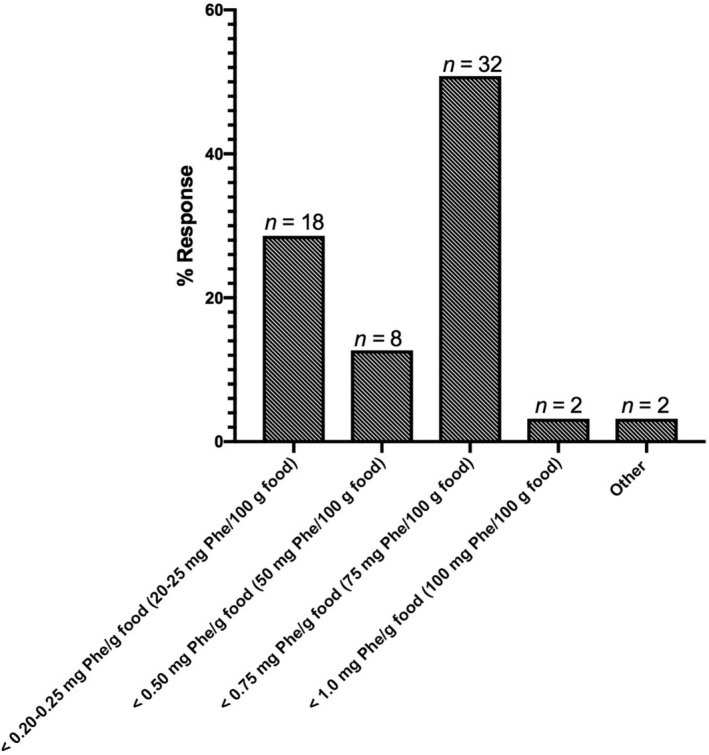
Phenylalanine thresholds for free/uncounted foods

Fifty‐two percent (n = 32/62) of survey respondents reported using a Phe threshold of <0.75 mg Phe/g food to define free/uncounted low‐Phe foods in their clinic. About 30% (n = 18/62) of respondents reported using a more stringent threshold of 0.20 to 0.25 mg Phe/g food, while 13% (n = 8/62) used a moderate threshold of 0.5 mg Phe/g food to define free/uncounted foods. Two respondents used a higher threshold of <1 mg Phe/g food; 2 additional respondents reported “other” (Figure 1).

Survey responses regarding protein thresholds were more variable; 60% of respondents (n = 37/62) reported that foods with <0.5 g protein/serving (roughly equivalent to 25 mg Phe/serving) were categorized as free/uncounted, and 21% (n = 13/62) reported that foods with <1 g of protein per serving were categorized as free/uncounted (Figure S1). Most survey respondents allowed patients to count either grams of protein or mg of Phe for foods that are counted/tracked (Figure S2).

Fifty‐eight percent of respondents (n = 36/63) reported confusion among patients regarding the term “free foods” (Figure S3).

### 3.3 Quantity limits for free/uncounted foods

Seventy‐one percent of survey respondents (n = 44/62) reported not restricting quantities of any free/uncounted food. An additional 27% of participants (n = 17/62) reported that they set limits on quantity for “some” or “all” free/uncounted foods (Figure S4). One survey respondent reported that they did not allow any free/uncounted foods, which was not in alignment with previous survey responses but could not be clarified further due to anonymity of participant responses.

Among participants who stated they restrict quantities of some or all free/uncounted foods, several mentioned obtaining diet records from patients and limiting quantities of frequently consumed foods. Bananas, dried fruit, orange juice, and modified low‐protein foods were among those foods most often defined as free/uncounted, but limited in total quantity per day.

### 3.4 Simplified Diet: age of introduction and patient exclusions

Sixty‐eight percent (n = 42/62) of survey respondents reported they would introduce the Simplified Diet approach at 6 months, which is when many infants in the U.S. start solid foods. An additional 26% of participants (n = 16/62) reported they would introduce the Simplified Diet at the earliest with children ages 12 months or older. Only 6% (n = 4/62) of survey respondents reported they would introduce the Simplified Diet at the earliest with children 5+ years or older, adolescents, or adults.

Survey respondents were queried whether there were any groups of patients with PKU for whom a Simplified Diet approach would not be appropriate; 68% (n = 42/62) reported that the Simplified Diet is appropriate for use with all patients. Eight percent of respondents (n = 5/62) felt that the Simplified Diet was not appropriate for individuals younger than 12 months, and an additional 11% (n = 7/62) felt this approach was not appropriate for individuals with classical PKU (defined in the survey as having pretreatment blood Phe concentrations >1200 umol/L). Finally, 13% (8/62) felt this diet approach was not appropriate for women with PKU during pregnancy (maternal PKU).

### 3.5 Clinician perceptions of the Simplified Diet

**Figure 2:**
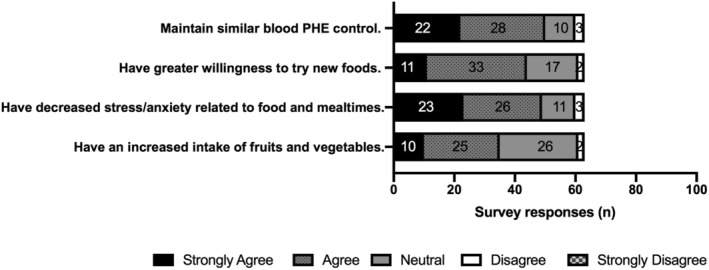
Clinician responses to survey prompt, “PKU Patients following a Simplified Diet…”

Seventy‐eight percent (n = 49/63) of survey respondents “agreed” or “strongly agreed” that patients following a Simplified Diet have decreased stress or anxiety related to food and mealtimes, and 79% (n = 50/63) “agreed” or “strongly agreed” that patients on a Simplified Diet maintain similar blood Phe control compared to patients following a diet where all foods are measured/counted (Figure 2). There was less agreement about whether those following a Simplified Diet have an increased intake of fruits and vegetables, or have a greater willingness to try new foods.

### 3.6 Implementation of the Simplified Diet: challenges

Sixty‐five percent of survey respondents (n = 41/63) reported that a patient/caregiver's lack of interest in switching from a traditional counting approach was a barrier to implementing the Simplified Diet. Forty‐six percent (n = 29/63) identified lack of educational materials as a challenge in Simplified Diet implementation. Other challenges/barriers included lack of consensus for defining free/uncounted foods (35%, n = 22/63), lack of published research on the Simplified Diet (22%, n = 14/63), and lack of acceptance from other medical team members (14%, n = 9/63).

## 4 DISCUSSION

This survey describes the clinical experience of dietitians in U.S.‐based metabolic centers related to use and implementation of the Simplified Diet for PKU. Almost all survey respondents reported using some version of the Simplified Diet for PKU nutrition management, reflecting widespread clinician interest in this approach. The survey identified areas of strong agreement, such as the age of introduction of the Simplified Diet at 6 to 12 months, as well as areas of widespread practice variability, such as specific Phe or protein thresholds used to define free/uncounted foods and whether or not to set daily quantity limits on these foods.

A major point of variation among dietitians was the precise definition of free/uncounted foods, which are those foods low in Phe that can be eaten freely without measuring or tracking. The majority of survey respondents reported using a Phe threshold of <0.75 mg/g food to define free/uncounted foods; this threshold has been reported previously,^10^ and is slightly more liberal than the common thresholds for free/uncounted foods reported in European metabolic centers.^12^ Results from this survey may reflect the influence of studies published since 2009 showing that patients maintain good metabolic control on Simplified Diets with higher Phe thresholds for free/uncounted foods.^9^, ^10^, ^11^ Almost a third of respondents used a lower threshold of 20 to 25 mg Phe/g food, which allows only a small number of fruits, vegetables, and low‐protein medical foods to be eaten without tracking, and may offer limited advantages to patients over the traditional approach. There was also significant variation in dietitians' thresholds for the amount of protein per serving, typically listed on a standard Nutrition Facts Label, considered free/uncounted.

Most respondents reported not restricting the quantity of free/uncounted foods, indicating some agreement that these foods, however defined, should be allowed in unrestricted amounts. Further research and discussion is needed to establish a consensus Phe and protein threshold for free/uncounted foods that goes beyond fruits, vegetables, and low‐protein medical foods to encompass commercially prepared foods with a Nutrition Facts Label.

Survey respondents reported significant confusion among patients regarding the term “free foods,” possibly related to these differences in clinic guidelines. Further, patients may not fully appreciate that metabolic dietitians adjust Phe/protein prescriptions to account for a certain amount of their total Phe from free/uncounted foods. Although these foods are not counted or tracked by the patient/caregiver, they are accounted for in the overall diet prescription. Dietitians may wish to revisit word choice for foods that do not need to be counted towards the daily Phe or protein requirement, such as “uncounted” foods or another term that reflects these foods more accurately within the Simplified Diet. Standard language and consistent guidelines on free/uncounted foods across metabolic centers may decrease patient confusion.

While most survey respondents felt the Simplified Diet was appropriate for all patients starting from 6 to 12 months of age, several participants noted that they would not use a Simplified Diet approach with patients whose blood Phe routinely exceeded goal treatment ranges. While some patients are sensitive to small variations in Phe intake, the Simplified Diet approach can be customized to meet the needs of patients with wide ranges of phenylalanine tolerance by lowering the amount of “counted” Phe/protein while maintaining a wide variety of free/uncounted foods. Existing research suggests that the Simplified Diet maintains equivalent metabolic control compared to a traditional diet^7^, ^8^, ^9^, ^10^, ^11^; however, there is no published data on the degree of metabolic control attained with the Simplified Diet in women with PKU during pregnancy, or in children younger than 2 years of age.^9^, ^10^, ^11^

This study had several limitations. It was a cross‐sectional, nonvalidated survey, although the questionnaire was tested and revised extensively by experienced metabolic dietitians. Multiple survey submissions per clinic were allowed, so survey findings represent the perspectives of individual dietitians rather than a center‐based approach. The questionnaire was sent out via an email listserv, and participants were self‐selected; it is possible that dietitians who use the Simplified Diet approach in clinical practice were more likely to respond to the survey invitation. This study also had several strengths, including geographical representation from dietitians across the U.S. and U.S. territories and with a range of experience in metabolic nutrition.

### 4.1 Future education and research needs

Survey respondents identified a need for additional Simplified Diet educational materials, including patient‐oriented resources in multiple languages on serving sizes, nutrition labels, and meal planning, as well as online resources for tracking food intake. One Simplified Diet tracking tool is already available through http://howmuchphe.org.

Future research is needed to evaluate the patient experience with the Simplified Diet compared to traditional counting methods, and to determine how many patients are utilizing this approach. In addition, the effects of the Simplified Diet on metabolic control in women with PKU during pregnancy and in children younger than 2 years of age are important areas for future study. Finally, development of clinical practice guidelines may promote a more standardized approach towards the Simplified Diet, allowing for comparability across centers.

### 4.2 Conclusions

This survey aimed to better understand current clinical experience related to use and implementation of the Simplified Diet for PKU among dietitians in U.S.‐based metabolic centers. The findings from this survey provide an overview of the range of current practice, and represent an important first step towards a more standardized Simplified Diet approach.

## CONFLICT OF INTEREST

The authors declare that they have no conflict of interest.

## AUTHOR BUTIONS

J.G.H. designed the project, obtained funding, and collected the data. J.G.H., S.H., N.D., S.V.C., F.R., and L.B. contributed to survey development. J.G.H. analyzed the data; J.G.H., S.H., N.D., S.V.C., F.R., and L.B. contributed to interpretation of the data and development of the paper. J.G.H. wrote the paper with significant input from all authors.

## INFORMED CONSENT

This study was approved by the Oregon Health & Science University Institutional Review Board, Portland, Oregon. All procedures followed were in accordance with the ethical standards of the responsible committee on human experimentation (institutional and national) and with the Helsinki Declaration of 1975, as revised in 2000 (5). Informed consent was obtained for each survey participant.

## ANIMAL RIGHTS

This article does not contain any studies with animal subjects performed by any of the authors.

## Supporting information


**Supplemental Figure S1** Survey respondents that limit the quantities of free/uncounted foods in the Simplified Diet.
**Supplemental Figure S2** Survey respondents recommended tracking tool for patients who are following the Simplified Diet.
**Supplemental Figure S3.** Survey respondents who felt there is confusion among PKU patients about the term “free” foods.
**Supplemental Figure S4.** Survey respondents that limit the quantities of free/uncounted foods.
