# Supplementary material for: Simplified Diet for nutrition management of phenylketonuria: A survey of U.S. metabolic dietitians
Source: JIMD Rep. 2020 Apr 8;53(1):83–9. doi: 10.1002/jmd2.12106 (PMC7203646; doi:10.1002/jmd2.12106)

**SUPPLEMENTARY MATERIAL**

**Supplemental Figure 1.** Survey respondents that limit the quantities of free/uncounted foods in the Simplified Diet.


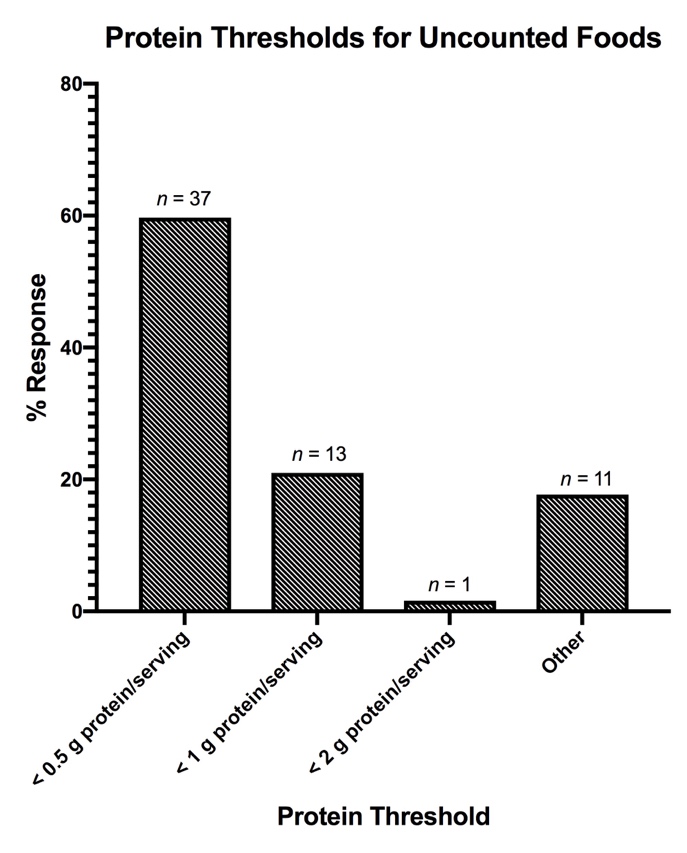


**Supplemental Figure 2** Survey respondents recommended tracking tool for patients who are following the Simplified Diet.


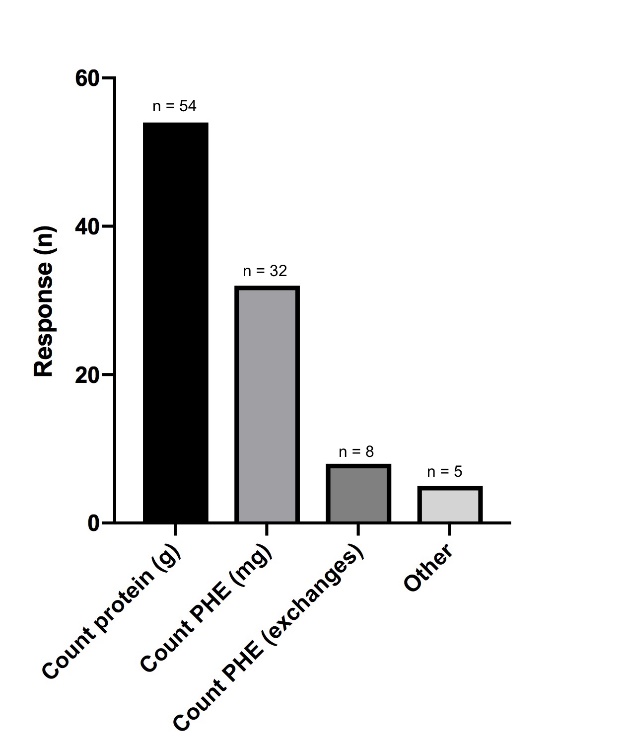


**Supplemental Figure 3.** Survey respondents who felt there is confusion among PKU patients about the term “free” foods.


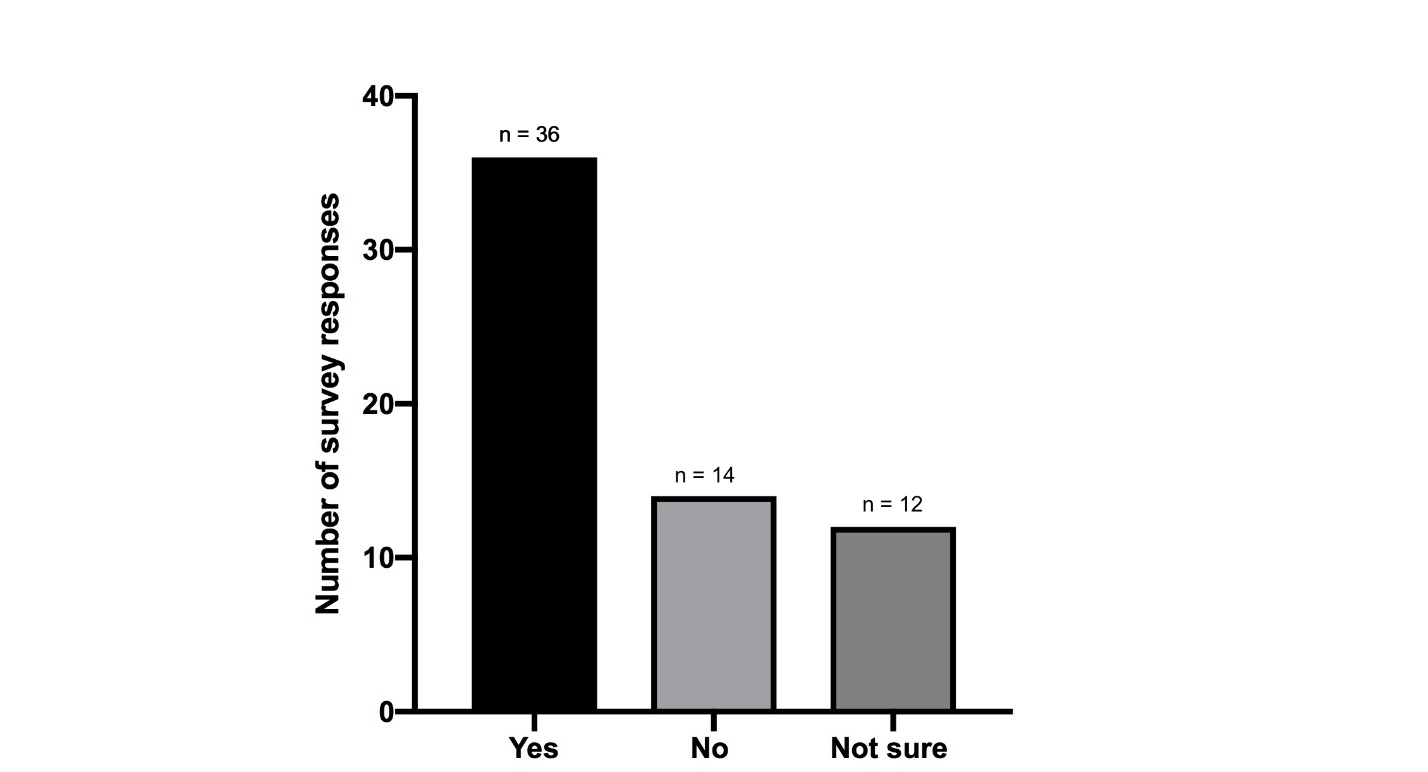


**Supplemental Figure 4.** Survey respondents that limit the quantities of free/uncounted foods.


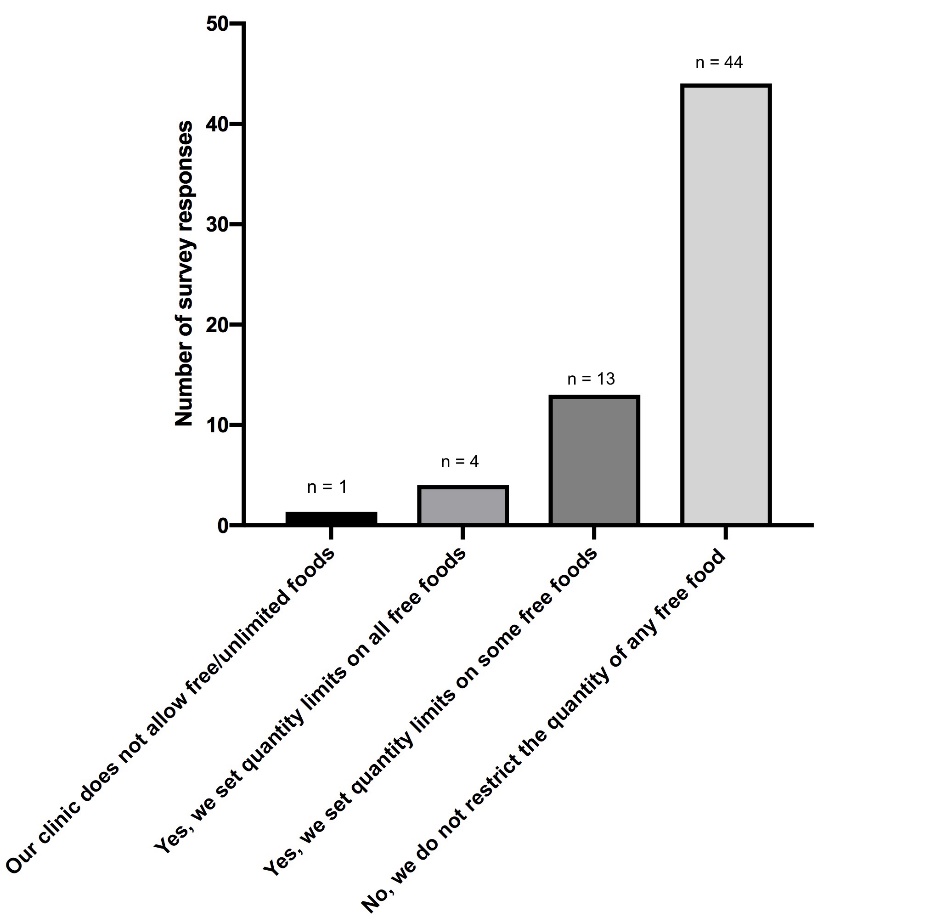

Supplement: Supplementary file 1 — Supplemental Figure S1 Survey respondents that limit the quantities of free/uncounted foods in the Simplified Diet. Supplemental Figure S2 Survey respondents recommended tracking tool for patients who are following the Simplified Diet. Supplemental Figure S3. Survey respondents who felt there is confusion among PKU patients about the term “free” foods. Supplemental Figure S4. Survey respondents that limit the quantities of free/uncounted foods. [file JMD2-53-83-s001.docx]
